# Trajectory optimization and obstacle avoidance of autonomous robot using Robust and Efficient Rapidly Exploring Random Tree

**DOI:** 10.1371/journal.pone.0311179

**Published:** 2024-10-11

**Authors:** Naeem Ul Islam, Kaynat Gul, Faiz Faizullah, Syed Sajid Ullah, Ikram Syed

**Affiliations:** 1 Department of Computer Science and Engineering and (IBPI), Yuan Ze University, Taoyuan City, R.O.C (Taiwan); 2 National University of Science and Technology, Islamabad, Pakistan; 3 Department of Information and Communication Technology, University of Agder (UiA), Kristiansand, Norway; 4 Dept Information and Communication Engineering, Hankuk University of Foreign Studies, Yongin, South Korea; University of Hull, UNITED KINGDOM OF GREAT BRITAIN AND NORTHERN IRELAND

## Abstract

One of the key challenges in robotics is the motion planning problem. This paper presents a local trajectory planning and obstacle avoidance strategy based on a novel sampling-based path-finding algorithm designed for autonomous vehicles navigating complex environments. Although sampling-based algorithms have been extensively employed for motion planning, they have notable limitations, such as sluggish convergence rate, significant search time volatility, a vast, dense sample space, and unsmooth search routes. To overcome the limitations, including slow convergence, high computational complexity, and unnecessary search while sampling the whole space, we have proposed the RE-RRT* (Robust and Efficient RRT*) algorithm. This algorithm adapts a new sampling-based path-finding algorithm based on sampling along the displacement from the initial point to the goal point. The sample space is constrained during each stage of the random tree’s growth, reducing the number of redundant searches. The RE-RRT* algorithm can converge to a shorter path with fewer iterations. Furthermore, the Choose Parent and Rewire processes are used by RE-RRT* to improve the path in succeeding cycles continuously. Extensive experiments under diverse obstacle settings are performed to validate the effectiveness of the proposed approach. The results demonstrate that the proposed approach outperforms existing methods in terms of computational time, sampling space efficiency, speed, and stability.

## 1. Introduction

Nowadays, transportation and industry frequently use portable robots addressed by autonomous vehicles [1–3]. Autonomous vehicles include a framework for perception, navigation, and control, and the study of the robot organizing framework has always been a major concern. One of the main challenges is how to produce a proper path to reach a target location without collisions for autonomous vehicles [4–6]. Geometric search techniques and graph search methods have been explored in earlier research [7]; the computations of geometric approaches are extremely straightforward, useful, and reasonable, such as spline curves, and they adapt well to basic environments. Even though geometric approaches offer certain benefits, they have notable drawbacks. These path-planning techniques need improved intelligence and adaptability in complex contexts [8].

The effectiveness of optimization techniques frequently employed in mobile robot route planning has been demonstrated while seeking paths based on graphs. Forward searching in continuous coordinates is typically carried out using RRT [9]. Although this technique performs quick searches, it is not suitable for confined spaces when the situation is complicated. The A* algorithm can discover the shortest obstacle-free path based on specific decision criteria [10]. However, the resultant path often consists of difficult-to-follow straight lines. Significant efforts have been made to improve the performance of the A* method. For instance, the Jump Point Search algorithm can speed up the A* method by an order of magnitude [11]. Liu et al. [12] extended the Jump Point Search technique from a two-dimensional to a three-dimensional environment. By including dynamic constraints, the Hybrid A* algorithm [13] can create smooth paths that accommodate the robots. The graph-based approach can identify an optimal path if a viable path already exists; if not, it will result in failure. This demonstrates the completeness and resolution excellence of the graph-based approach. However, because the search space created by the graph-based deconvolution of the state space is too large, graph-based techniques cannot effectively address large-scale problems (such as those involving industrial robotic arms). The D* algorithm was designed to guide autonomous vehicles in a two-dimensional space. Its main advantage is that it can choose the best course while navigating a difficult environment [14]. However, this method is typically constrained by vehicle kinematics.

The capacity of various research units to plan paths was demonstrated during the 2007 Defense Advanced Research Projects Agency (DARPA) Urban Challenge. The proposed plans and theoretical models laid the groundwork for further investigation. The “Talos” vehicle, created by MIT, employed a closed-loop RRT-based path planning technique [15]. Dolgov et al. [16] developed a well-hybrid A* searching technique, which uses the vehicle’s 3-dimensional kinematic state space and local planning via nonlinear optimization, leading to a local optimum. However, these path-planning techniques have not proven their viability and efficacy in complex, challenging environments. In past years, Suresh et al. [17] employed FSVM to ensure an accident-free route while avoiding several dynamic obstacles. The outcomes indicate that this approach is successful. Simulations using this method, which generates fuzzy rules from plain evaluation data, are shown. Chu et al. [18] implemented an algorithm for real-time route planning, focusing on selecting the safest and simplest path after creating multiple pathways based on predefined checkpoints. Makarem and Gillet [19] designed a steering component appropriate for autonomous vehicles, though it does not consider the impact of obstacles. Chebly et al. proposed the “tentacle method” [20], which creates a series of virtual spines showing potential routes for the vehicle, with the optimal route selected based on an evaluation function. Moreau et al. [21] developed a better curve design approach for complex, dynamic environments. This approach considers all sensors required for obstacle detection and transforms the planning problem into an optimal problem, solved using Lagrangian and gradient-based methods. Tazir et al. [22] use two strategies for real-time planning: Dijkstra’s algorithm and genetic algorithms for avoiding static obstacles, and the wait/accelerate principle for travelling in dynamic areas. Although this approach is test-efficient, it does not consider robot kinematic restrictions. Researchers have proposed an innovative Integrated Local Trajectory Planning and Tracking Control (ILTPTC) framework to allow automated vehicles to travel along a basic track while avoiding detection and meeting vehicle kinematic limitations [23]. This architecture employs an MPC-based planning technique, which can meet vehicle kinematics requirements but falls short of actual needs. The sampling-based planning method is a crucial planning methodology. Compared to discretizing the state space, sampling-based planning generates a graph or tree by selecting random points in the state space. In large-scale applications, sampling-based planning algorithms perform better than graph-based planning algorithms. Since the sampling-based planning method is stochastically complete, the probability of finding a suitable path approaches 1 as the number of samples approaches infinity. Two important sampling-based planning techniques are RRT [24] and Probabilistic Roadmaps (PRM) [25]. PRM, a multi-query motion planning method, produces a viable path after generating a feasible graph reflecting the spatial connection through random sampling in the state space. PRM can be used to search for various pathways after creating the graph, but mapping out the entire area for a single search requires significant effort. RRT, being a one-request route planning technique, is quicker than PRM. It builds a tree with its root at the starting point and searches the state space by randomly generating states, selecting the nearest random tree nodes, and growing the random state from the nearest neighbour selection point. The search is accomplished when the tree reaches the desired location, and RRT retraces its steps to find a workable route. RRT can quickly identify an initial path in a high-dimensional area, although it has several flaws. For instance, due to random sampling, the variance of its runtime is substantial, which means it can take a considerable amount of time to find a suitable route. Additionally, RRT does not perform well in environments with narrow passages [26]. Furthermore, since the path is generated randomly, it is likely that the path found using RRT is not optimal [27]. Rapidly Exploring Random Tree Star or RRT*, is considered a significant improvement on RRT [28]. RRT* continues to optimize the original path after discovering it by continuously sampling [29]. To determine the optimal path, RRT* incorporates neighbor-searching and rewiring tree processes. It is demonstrable that, with an infinite number of samples, the path generated by RRT* is optimal. However, RRT* requires a significant amount of memory and time to determine the optimal path [30]. Like RRT, RRT* is also affected by substantial search time volatility.

Significant efforts have been made to improve the quality of the pathways identified by RRT and RRT*. For instance, Kino dynamic RRT* [31] can achieve an optimal route that meets static constraints by extending RRT* to Kino dynamic systems. Anytime-RRT* allows for rapid path re-planning from any location. Another focus area in RRT-related research is increasing the search rate and reducing search time variance. For example, RRT-Connect [32] builds two trees, one rooted at the starting state and the other at the destination state, then moves the two trees towards each other. A 2D Gaussian mixture model is used in [33] to quickly find a good initial solution. The training dataset in this article, which includes map data and the best route, is created using the A* algorithm [34]. Batch Informed Trees [35] swiftly locate a viable path by restricting the state space to a gradually growing subset. However, these techniques mainly work effectively in specific settings. RRT can speed up the search process when combined with various path search strategies. For example, the artificial potential field (APF) technique is incorporated into RRT* in [36] to accelerate the convergence rate, although planning time may rise significantly in complex situations. The efficiency of the path-tracking controller is verified using MATLAB to simulate the car depicted in ADAMS. To assess the interaction between real-time planning and tracking control of smart cars, Zhou W [37] recommended new infrastructure that relies on an upgraded RRT method and a linear time-varying (LTV) path planning and tracking control. Based on the LTV-MPC method, the basic RRT algorithm is modified to ensure the intended course complies with the vehicle’s kinematic constraints and approaches the optimal outcome. These modifications include target orientation, node pruning, curve fitting, and optimal path selection. The impact of variables, including vehicle speed, planning step, and cycle on real-time planning and stability tracking, is then examined. Mata S proposes a vehicle path planning approach based on a simple linear and time-invariant monorail framework computed using a uniform nominal longitudinal speed. To compensate for variations in dynamic systems between the actual vehicle and this constant nominal model, a tube-based resilient model predictive control (MPC) strategy is presented [38].

The A*-RRT* technique [39] significantly speeds up convergence by using the route created by the A* method to guide the RRT* planner’s sampling process. However, for complex problems, A* requires a considerable amount of effort to identify a starting point. While LM-RRT [40] uses reinforcement learning techniques to direct tree development, learning-based approaches might not function effectively in novel environments. It has been demonstrated that curve interpolation is an effective method for creating reference paths. Many researchers have employed polynomials [41], Bezier curves [42], and B-splines [43]. To increase the autonomy of the vehicle and decrease the number of turns on the planned course, an adaptive ant colony optimization (ACO) path planning approach is applied [44]. One of the most important aspects of autonomous navigation is collision avoidance, where the robot must find its way from the starting point to the destination while avoiding obstacles. Extensive research has been done in the area of collision avoidance, including [45–53]. Furthermore, in recent years, motion planning research has made extensive use of learning-based methodologies. Neural RRT* uses deep learning to discover a probability distribution for sample selection. RL-RRT investigates a deep reinforcement learning strategy as a local planner and employs a distance function that trains through deep learning to bias tree growth toward the targeted area. An approach that combines inverse reinforcement learning with RRT* is used to learn the cost function of RRT* [54]. The DL-P-RRT* method uses a virtual artificial potential field to understand the function of the artificial potential field before applying it to the RRT* algorithm. Learning-based approaches work well in specific situations, but they may struggle to generalize in unfamiliar environments.

Furthermore, a bidirectional RRT-type motion planning algorithm for hybrid systems, called HyRRT-Connect, is proposed in [55]. This algorithm constructs two search trees for motion planning: the first tree starts from the initial point and propagates in the forward direction, while the second tree begins at the goal point and propagates in the backward direction. Once an overlap between the forward and backward paths is detected, a connection is established between them. While HyRRT-Connect ensures a path from the source to the destination, it doubles the search time, whereas the proposed approach converges faster. In [56], the DT-RRT* algorithm is introduced, combining the double-tree structure with RRT*. This method also employs two trees: one for space exploration and the other for optimization. Although this algorithm performs well, it requires additional searches. A novel optimal path planning algorithm based on convolutional neural networks (CNN), called neural RRT* (NRRT*), is also proposed. The NRRT* algorithm utilizes a nonuniform sampling distribution generated from a CNN model and is trained using data samples from successful path planning cases. While effective, this algorithm requires data samples for training the neural network models, which can be difficult to obtain in some scenarios.

To address the flaws in path tracking found in traditional methods like RRT and RRT*, R. Mashayekhi [57] introduced Informed RRT*-Connect, which decreases the number of searches compared to previous methods. However, our proposed RE-RRT* expedites the search for the optimal route while limiting the number of randomly generated search nodes and minimizing the convergence rate. It generates random nodes only near obstacles and proceeds along a single path toward the goal position within 9 to 10 seconds. Therefore, RE-RRT* is quicker than Informed RRT*-Connect when searching for a path that is close to optimal. These approaches often overlook efficiency gains achievable through targeted sampling and direct path integration, which RE-RRT* addresses comprehensively. This context highlights how RE-RRT* bridges gaps left by traditional and batch-based methods, offering superior performance in terms of convergence time and path quality.

In contrast to these algorithms, our Robust and Efficient RRT* (RE-RRT*) offers several additional functions and improvements. Unlike standard RRT*, which samples randomly across the entire space, RE-RRT* restricts sampling to areas near obstacles and along potential pathways to minimize unnecessary exploration. When a direct path from the start to the objective is possible, RE-RRT* reduces the need for significant tree expansion, thereby speeding up convergence compared to techniques like RRT-Connect. It employs a pruning technique to simplify and enhance the efficiency of the generated paths in complex scenarios with numerous obstacles.

Through focused sampling and efficient path extensions, RE-RRT* yields smoother path outputs and faster convergence rates compared to batch approaches such as Batch Informed Trees. While Neural RRT* emphasizes neural network-based steering and optimization, it requires data samples for training the neural network models, which can be challenging to obtain. In contrast, RE-RRT* prioritizes efficient tree growth and path refinement using traditional search techniques. These attributes make RE-RRT* a dependable and efficient path planning algorithm capable of functioning in challenging environments without compromising computational performance.

The remaining paper is divided into several sections. The second section provides the formal formulation of the motion planning problem and the relevant context. The third section defines the RE-RRT* approach proposed in this study. The forth section presents the simulation and assessment of our experimental findings. The final section concludes our work.

## 2. Problem statement

In this section, we outline the relevant background for this study. After introducing the formal concept of motion planning problems, we will discuss related algorithms such as RRT and RRT*.

Two primary challenges in path planning using RRT and RRT* are:

- convergence speed
- unnecessary sampling and searches

The following fundamental limitations in these methods still exist: (1) The use of random sampling lengthens the algorithm’s execution time and hinders convergence, and (2) The application of the nearest node selection technique frequently results in complicated scenario planning. (3) The planned path cannot be employed in the path planning of autonomous vehicles since it does not take vehicle kinematics restrictions into account.

**Fig 1:**
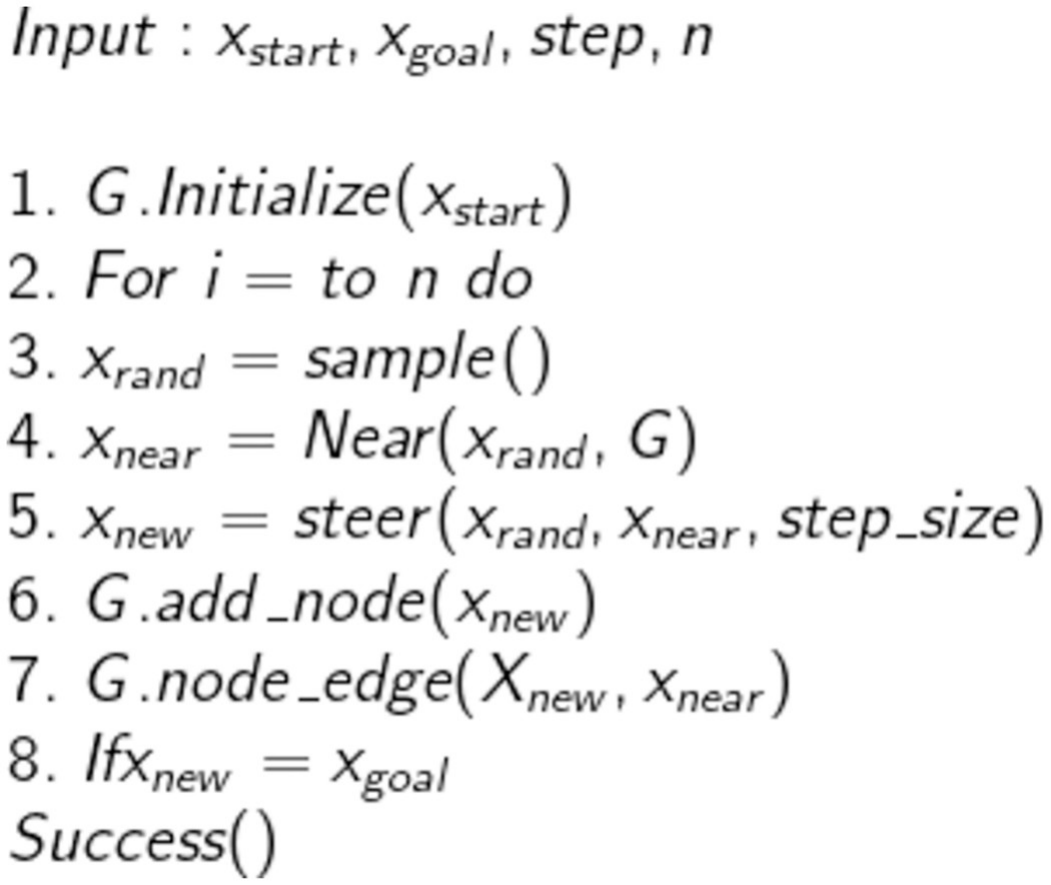
RRT algorithm.

**Fig 2:**
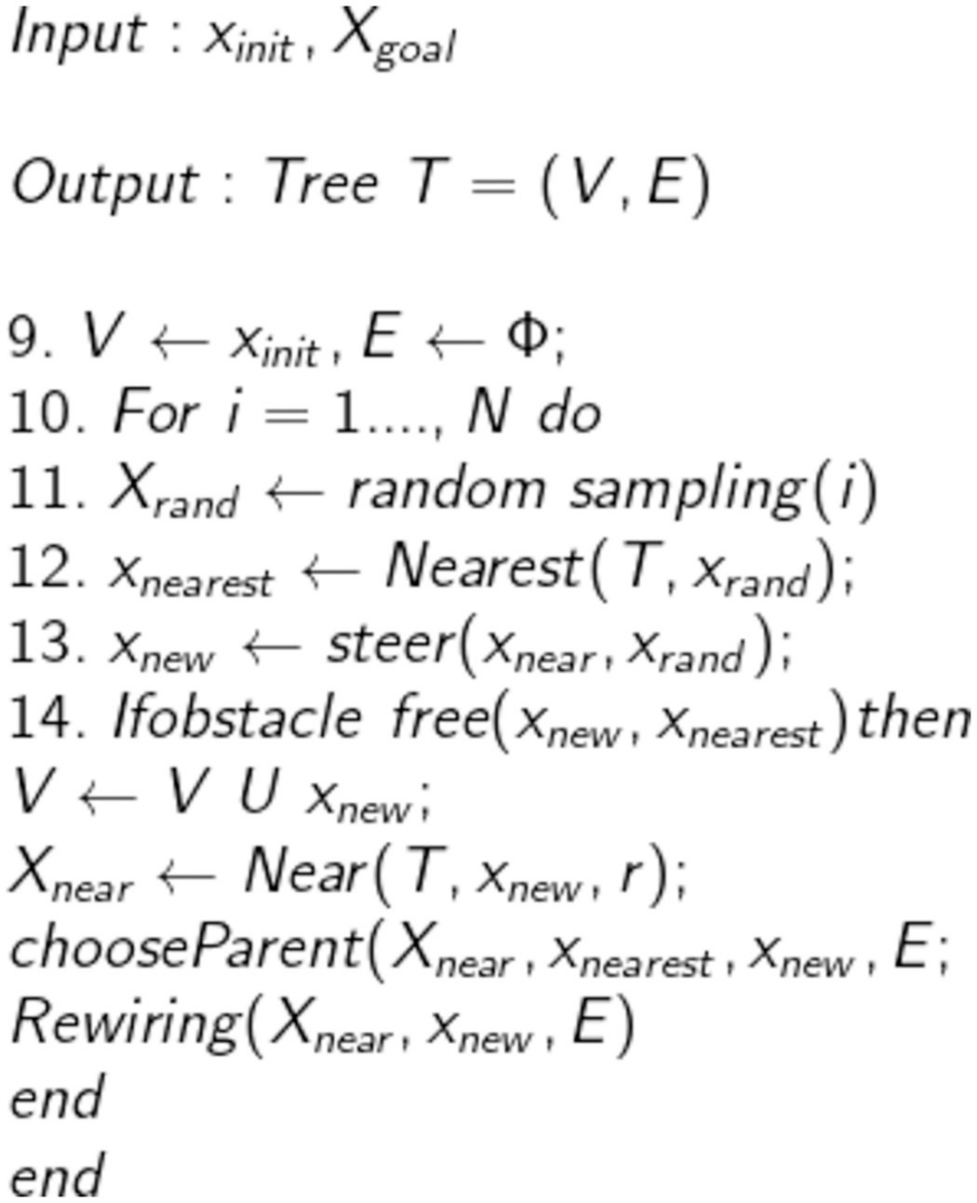
RRT* algorithm.

**Fig 3:**
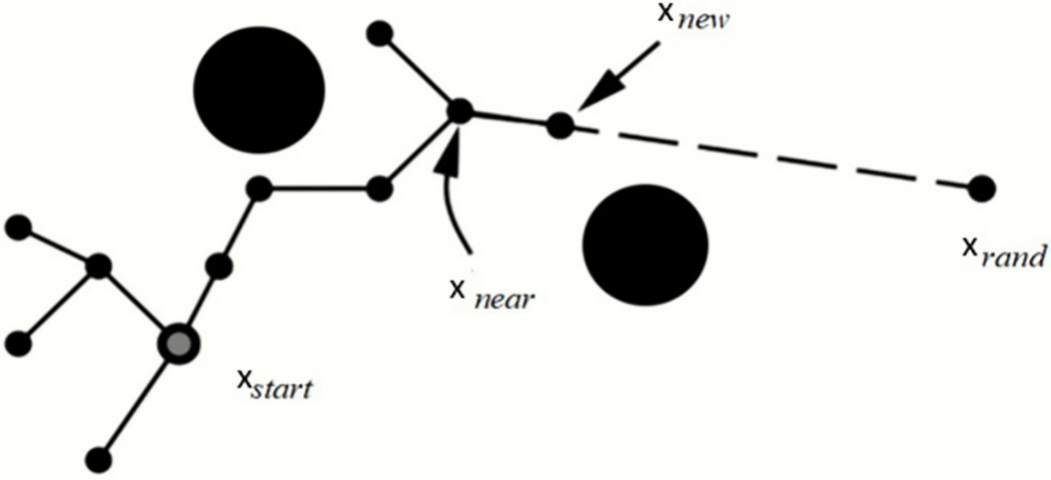
Schematic result of RRT algorithm.

The RE-RRT* method is based on the RRT algorithm, and RRT* is a crucial technique for determining the approximative optimum route. As a result, Algorithms 1 and 2 introduce the RRT and RRT* shown in Figs 1 and 2, accordingly. Lavalle formally presented the Rapidly Exploring Randomized Trees (RRT) approach in 1998. Beginning from the starting point and extending into a tree-like structure, the nodes of this method are mostly extended in the form of trees. Random sample locations in the planning area are used to decide the direction in which the form structure will expand. It can always identify a successful path regardless of how complex the environment is given enough time, making it probabilistically complete. Nevertheless, because random nodes are chosen each time to establish the search’s direction, the randomness is rather high, and the efficiency is low. The RRT is a technique built on a single query search that finds a viable path very rapidly. RRT creates a tree during the initiation step *x*_init_, with the starting state acting as the node. RRT chooses the closest vertex *x*_near_, after randomly sampling a state *x*_free_, in state space for each iteration. The steering function will then create the RRT algorithm which generates *x*_new_, as seen in Fig 3. If the edge c has no obstacles, then the set of nodes will be expanded by *x*_new_ and the set of edges will be expanded by { *x*_near_, *x*_new_} The search is completed if *x*_new_ found at the desired location *x*_goal_.

**A**. **DataSets**

The route that RRT chose might not be the best one. RRT* solves this issue by adding a rewire step. The best parent node for *x*_new_ will be found for nodes with a distance smaller than r surrounding it if the edge *x*_near_, *x*_new_ is free of obstacles. Additionally, RRT* considers *x*_new_ as a substitute parent node for existing nearby nodes in addition to adding it to the tree. Therefore, RRT* constantly modifies the random tree as the sample periods go closer to infinity until it discovers an ideal path. However, the RRT* takes a long time, making it unsuitable for systems that must immediately identify an optimal path. To minimize this issue, we suggest RE-RRT* which uses the RRT* algorithm with some addition that works efficiently and takes less time to converge to the goal position. The MATLAB trials demonstrate that the approach increases planning speed and success rate.

## 3. Methodology

We introduce our Robust and Efficient Rapidly Exploring Random Tree algorithm in this section. Section 3.1 presents the model structure of the suggested method. Sections 3.2–3.3 introduce further information.

### 3.1 Model structure

**Fig 4:**
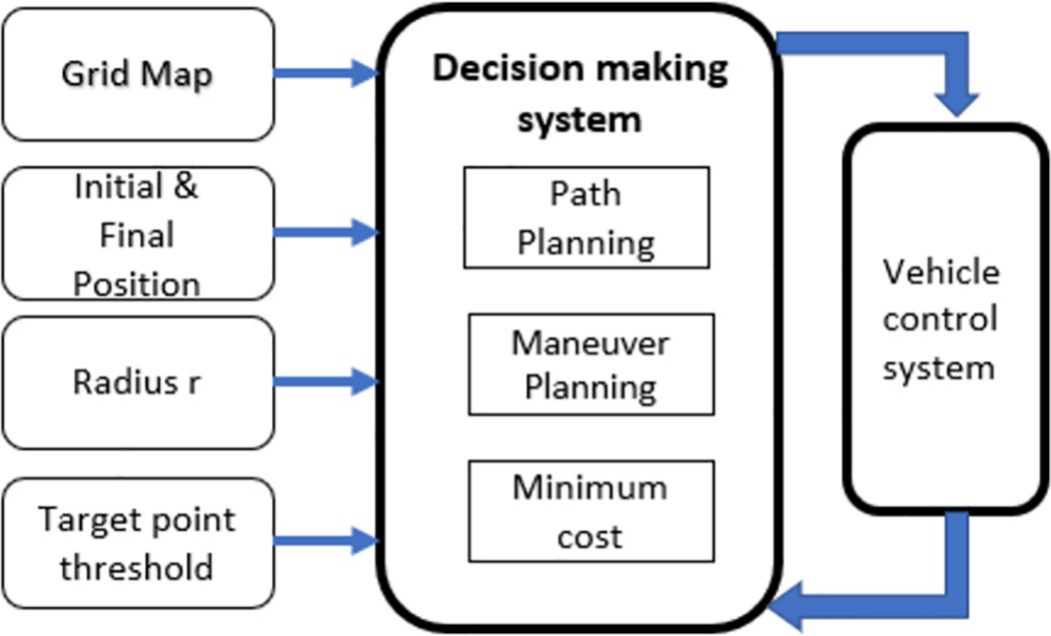
The RRT algorithm’s software architecture.

The decision-making system receives a grid map from the perception system. Global path, maneuver, and path-planning modules in the decision-making system work together to enable the vehicle to manage a variety of situations. The decision-making system then generates a route and sends it to the vehicle’s monitoring system. The suggested method’s model structure is shown in Fig 4. This model takes a grid map, which is m x n (here m x n can be any size like 800 x 800 or 500 x 500), and information about the initial position and goal position as input. Also, we set the target point threshold, expansion steps, rewire range, which is radius r, and maximum iteration. The entire grid map is filled with all the identified items. A collision-free route is swiftly generated using the steering constraint model.

**Fig 5:**
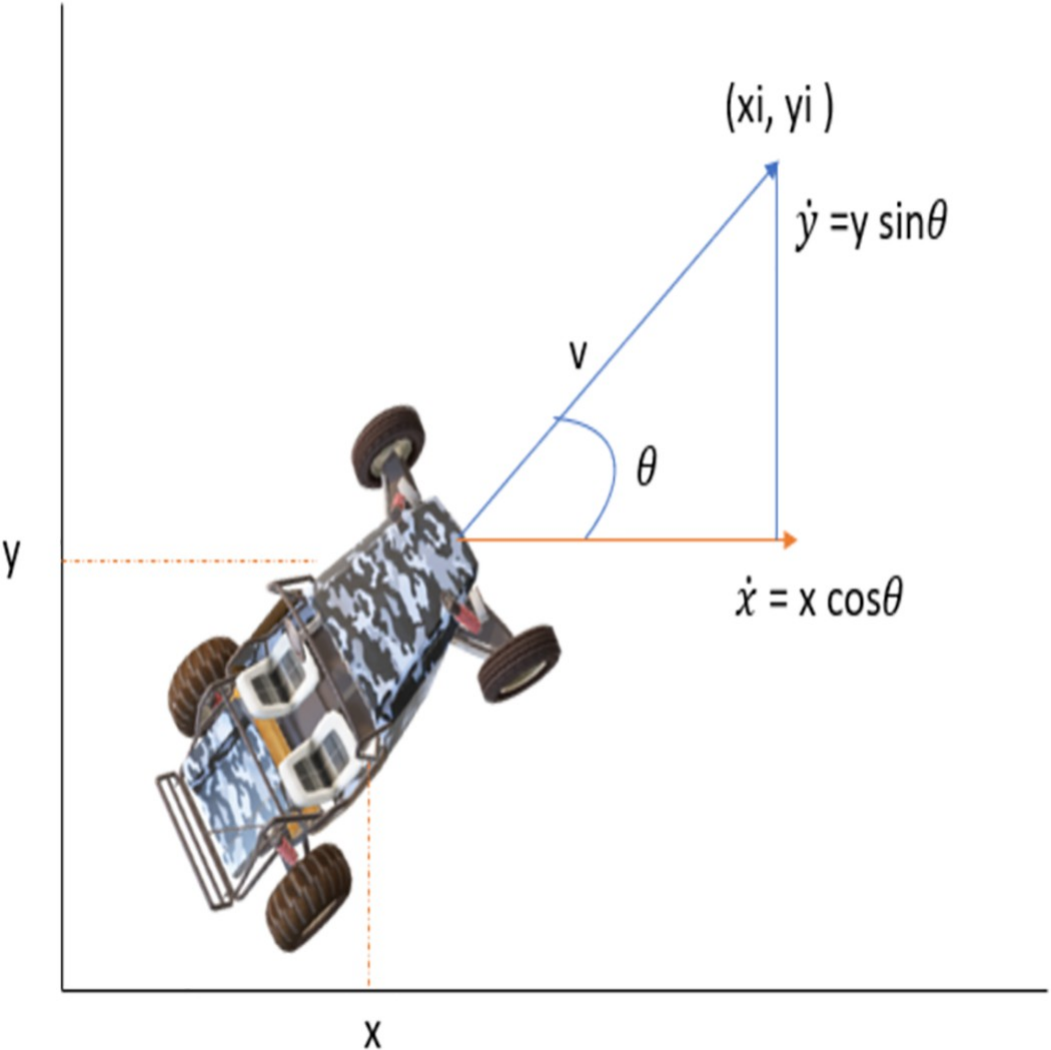
Vehicle kinematics model.

A simplified model of the vehicle is shown in Fig 5. The theta ‘*θ*’ that defines the orientation of the vehicle and the kinematic equation can be explained as follows:

$$\begin{array}{c} {}[\begin{array}{c} \dot{x}\\ \dot{y} \end{array}]=[\begin{array}{c} \text{cos}\;\theta \\ \text{sin}\;\theta \end{array}]v \end{array}$$ (1)

### 3.2 Framework of RE-RRT*

The complete framework and methodology of RE-RRT* is explained here. Which includes all steps from the start to the end of this algorithm. Our proposed algorithm is based on a sampling-based path-finding algorithm. A small difference between RRT* and RE-RRT* makes RE-RRT* more efficient and robust by limiting the random sampling and searches on the entire map. RE-RRT* limits unnecessary search, reduces the convergence time and makes the system efficient in this way.

#### 3.2.1 Improvement of RE-RRT*

The RE-RRT* algorithm has two major enhancements.

- Firstly, random sampling is limited, which helps to avoid searching over the entire space. In this way, our proposed algorithm speeds up the convergence rate.
- The second improvement is to minimize random nodes by limiting their generation only around the obstacles. Otherwise, our vehicle will be in a straight line from the initial position to the goal point.

**Fig 6:**
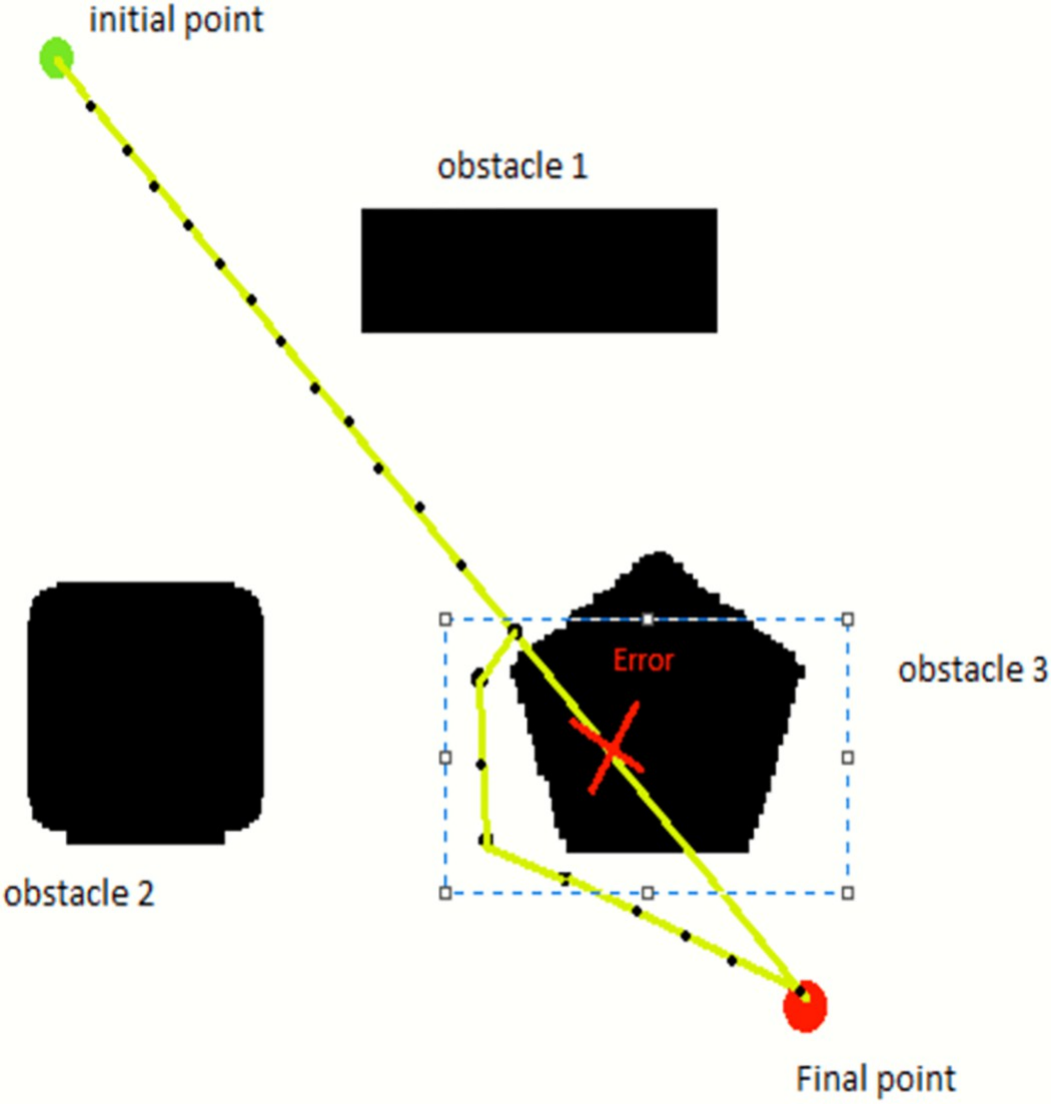
RE-RRT* algorithm’s searching schematic diagram.

One of the principles of the sampling-based path-finding algorithm is to build the search tree concurrently in the starting state and the goal state. The RE-RRT* initiative technique differs from the standard sampling-based path-finding algorithms, as shown in algorithms 1 and 2. From the start position, a path is generated in a straight line towards the goal position. After creating random spots across the obstacle, the tree is extended if there is a collision. After crossing the obstacle, the path leads to the goal position in a straight line until the next obstacle finds out on the vehicle path. RE-RRT* algorithm’s searching schematic diagram is shown in Fig 6.

Keeping in mind Fig 5, now we can visualize the RE-RRT* algorithm. The vehicle’s starting point is in green color and the ending point is in red. The huge black boxes are obstacles and we named them Obstacles 1, 2, and 3. Our vehicle started its path from the green signal moving forward towards the goal position in a straight line. When the vehicle reached near to the obstacle e.g., obstacle no 3, it stopped there. Until the sampling-based path-finding algorithm finds the path where there would be no obstacle and it would be a collision-free path. The blue dotted rectangle shows the area where there is random sampling and node generation as shown in Fig 3. This is the area from where the random tree generates and ends until it crosses the obstacle area. At that point, the random sampling and node generation process would stop and from that point, let’s say *x*_new_ to goal position straight line nodes will generate and our vehicle follow that path.

#### 3.2.2 The extension strategy of RE-RRT*

The extension strategy of RE-RRT* is a bit different from RRT*. Unlike RRT*, RE-RRT* limits unnecessary searches and reduces the sampling space by adding a line equation with RRT* method. The starting point of the vehicle is our starting node. The next nearest node in a straight line will be chosen by the given formula.

$$\begin{array}{c} \Delta_{x}=\frac{x_{\text{init}}-x_{\text{goal}}}{n} \end{array}$$ (2)

$$\begin{array}{c} \Delta_{y}=\frac{y_{\text{goal}}-y_{\text{init}}}{n} \end{array}$$ (3)

**Fig 7:**
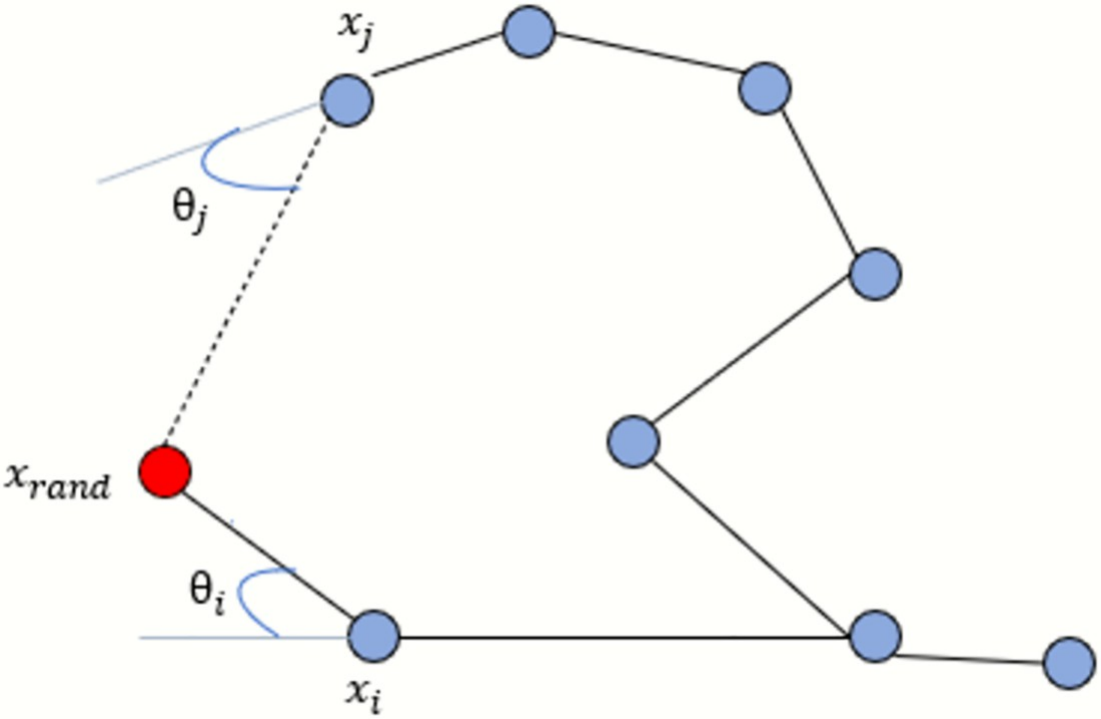
Schematic diagram for selecting the nearest node.

Here Δ_*x*_ and Δ_*y*_ are the nodes generating along the x and y-axis. The n defines the no of nodes which are generating on the straight path from the initial to the final position of the vehicle route. When the vehicle detects an obstacle on the path using the collision detection method of the RRT* algorithm, it stops and waits for the RRT* algorithm to generate a tree that navigates around the obstacle and finds an optimal path to cross it without colliding with it. At the beginning, the map is provided to the system, so the vehicle first scans the map to identify the obstacle areas. The collision detection algorithm uses the ceil and floor methods to check the height and width of the obstacle. Once the area of the obstacle has been scanned by the vehicle, then it can easily generate the random nodes across the obstacle and finds the shortest path to pass this area and move further towards the goal position. The tree generates the random nodes and rewires them as mentioned in Fig 7 to determine the shortest path and the starting nodes of the tree will be the coordinate (x,y) where our vehicle detects the obstacle existence and stops there. Using the Euclidean distance formula, we are looking for the closest nodes. Fig 7 shows how we select the nearest neighbour node by using the Euclidean distance formula.

The Euclidean distance between *x*_*i*_ and *x*_rand_ is closer, *θ*_*i*_ < *θ*_*j*_ so, *x*_*i*_ chosen as the closest neighbor node of the point which is under consideration. The parent node will be chosen based on whatever node produces the lowest cost to reach the random sample, and they are added appropriately. Following is a Euclidean distance equation that we used to determine the distance between the random sampling point and the nearby point:

$$\begin{array}{c} \text{Dist}=\sqrt{{\left(x_{\text{rand}}-x_{i}\right)}^{2}+{\left(y_{\text{rand}}-y_{i}\right)}^{2}} \end{array}$$ (4)

#### 3.2.3 Vehicle steering angle

By expanding the tree to get new nodes, there must be a factor theta, our vehicle steers according to it. *θ* is our vehicle’s steering angle.

$$\begin{array}{c} \theta =\text{atan2}(y_{\text{rand}}-y_{\text{near}},x_{\text{rand}}-x_{\text{near}}) \end{array}$$ (5)

$$\begin{array}{c} x_{\text{new}}=x_{\text{near}}+\text{cos}\left(\theta \right)\cdot \Delta_{x} \end{array}$$ (6)

$$\begin{array}{c} y_{\text{new}}=y_{\text{near}}+\text{sin}\left(\theta \right)\cdot \Delta_{y} \end{array}$$ (7)

Eqs 6 and 7 are written according to the vehicle’s kinematic model shown in Fig 5.

#### 3.2.4 Pruning process

The resulting pathways are typically exceedingly convoluted and uneven because of the sampling-based path-finding algorithm, especially in situations with numerous complicated obstacles. It is challenging to locate them successfully. The ride comfort of the vehicle will be impacted by too many fold points, which is unsuitable for path planning. Consequently, it is necessary to prune and smooth the routes of the sampling-based path-finding algorithm. To get a smooth and turn-free path, this algorithm deleted the useless nodes and updated them with useful nodes and the nodes that give optimal cost. It removes the number of turns in the path to achieve the pruning effect.

**Fig 8:**
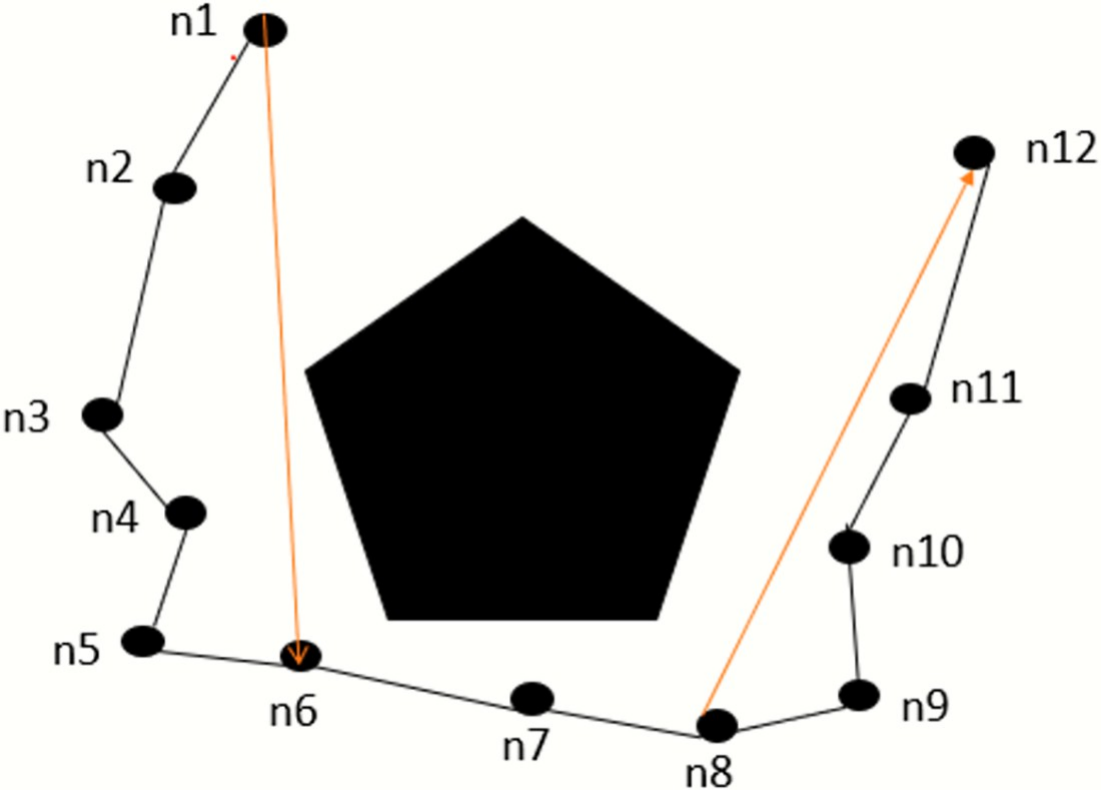
Pruning principle.

Fig 8 shows the pruning principle clearly. We have a solid obstacle and several random nodes generated around it. The irregular path sampling is shown in black lines. As we can see, the paths between nodes n1 to n6, and n8 to n12 are safe, so n1 to n6 and n8 to n12 can be connected directly, as shown in the red line. The next segment illustrates the contrast effect before and after pruning.

**Fig 9:**
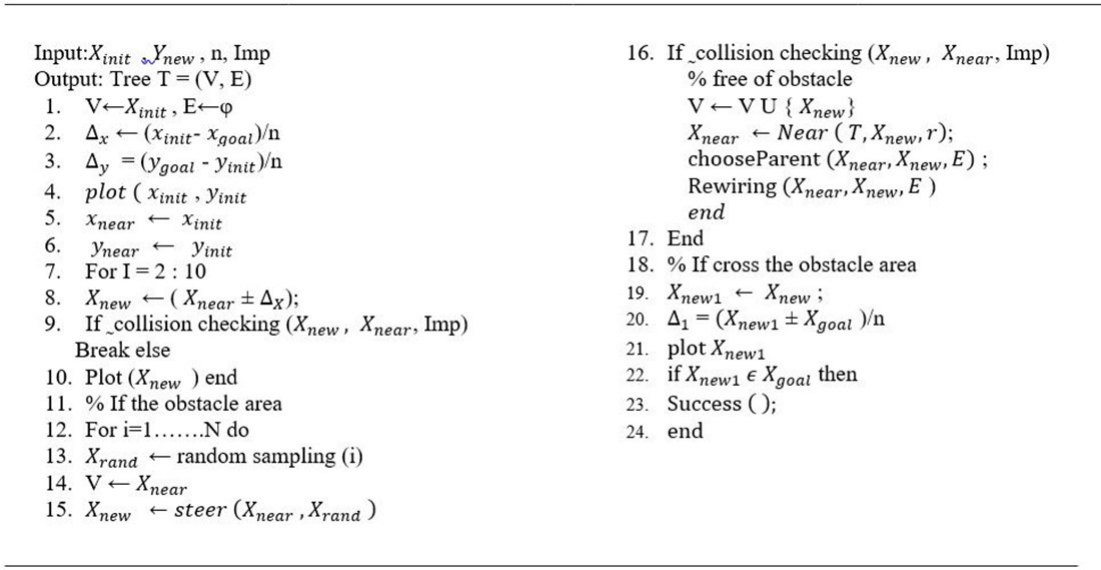
RE-RRT* algorithm.

To speed up the convergence rate of the vehicle, two main improvements, which we discussed above, are mentioned in Fig 9. This is the complete pseudo-code for robust and efficient rapidly exploring random tree star (RE-RRT*).

**Fig 10:**
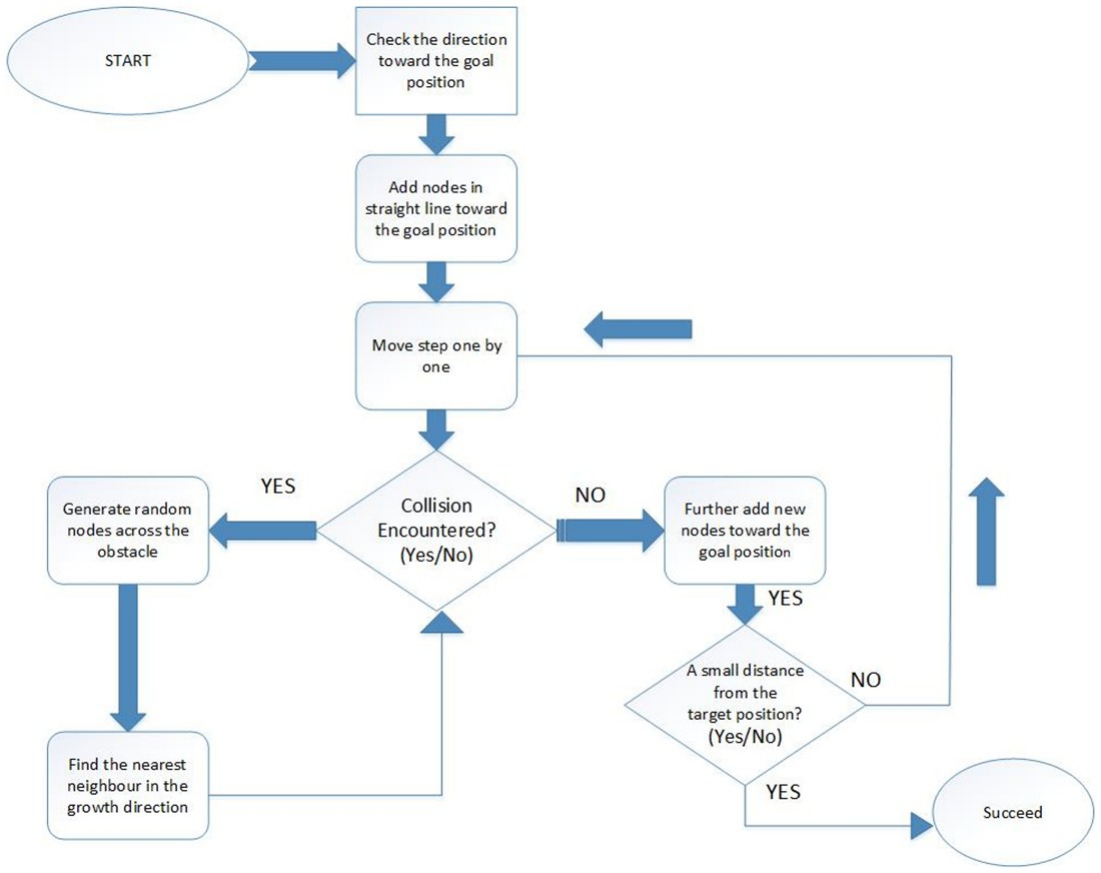
Flow chart of RE-RRT* algorithm.

Fig 10 is a schematic representation of the RE-RRT* method flow chart. It entails the following steps: It starts from the initial points and then checks the direction towards the goal position. Nodes will be added in a straight line. Moves steps one by one. When there is an obstacle on the way, random nodes will be generated around the obstacle until the vehicle reaches the goal position. Besides this, it also checks the neighbor nodes and updates the shortest nodes by deleting the previous ones.

## 4. Simulation and results

In this research, MATLAB R2022a is utilized for the simulation experiment, and the size of the experimental map is m X n. There are various obstacles throughout the map. The vehicle navigates the obstacles as it goes from its starting position to its destination location. Table 1 displays the algorithm’s parameter settings. We include obstacles in the map to test the performance of RE-RRT* in complicated spaces. Several tests were carried out to assess the performance of the suggested method, and we eventually compared the outcomes between previous sampling-based path-finding algorithms.

**Table 1:** The hyperparameters setting of RE-RRT*.

Hyper Parameters	Values
Size of image	m x n
Start Point	(*X*_init_,*Y*_init_)
Goal Point	(*X*_goal_,*Y*_goal_)
Segments on the line	10-15
Radius for neighbor node	30.0
Expansion step	30.0
Max Iteration	1000
Update time	50.0
Delay Time	0.0

**Fig 11:**
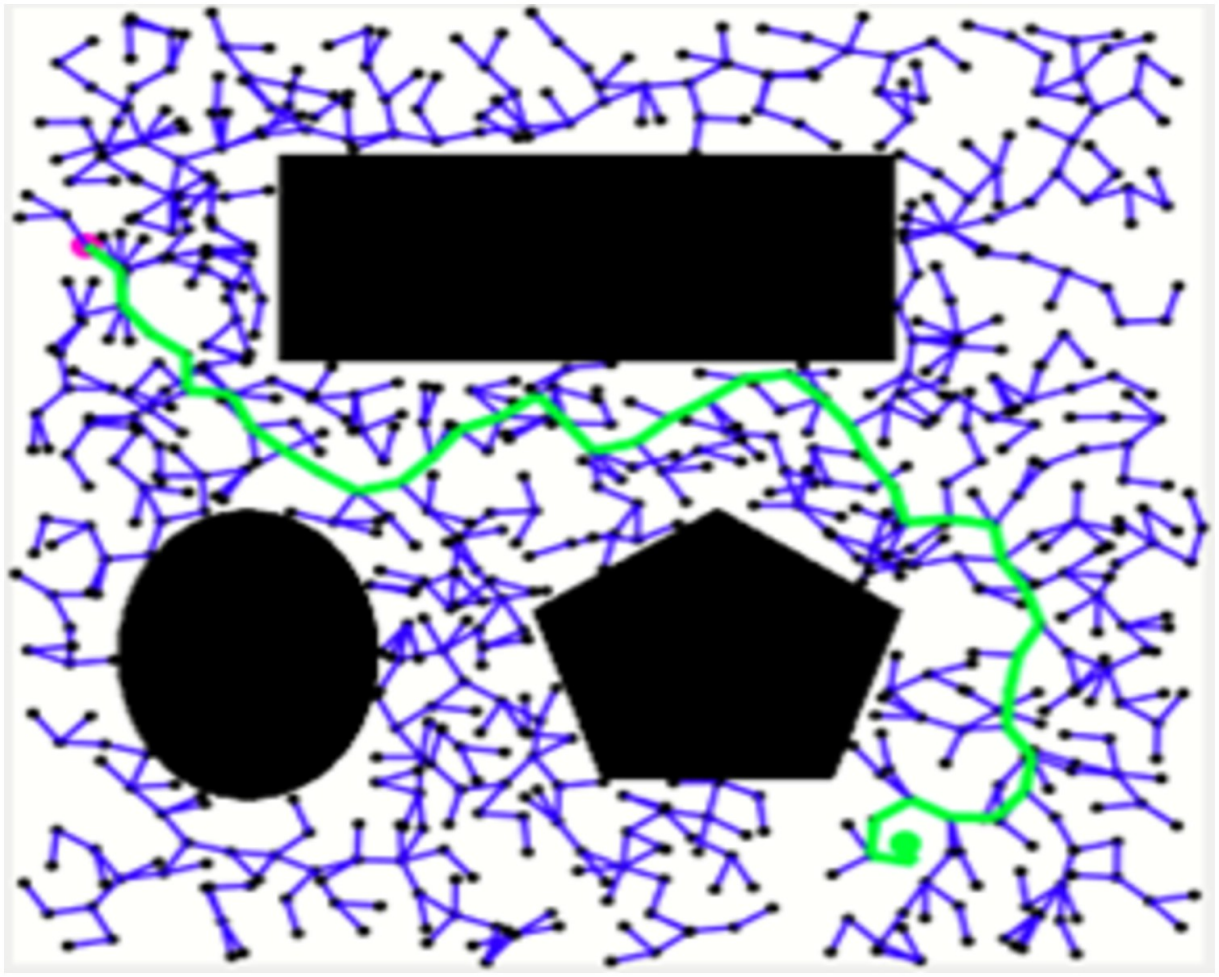
The result of RRT for experiment 1.

**Fig 12:**
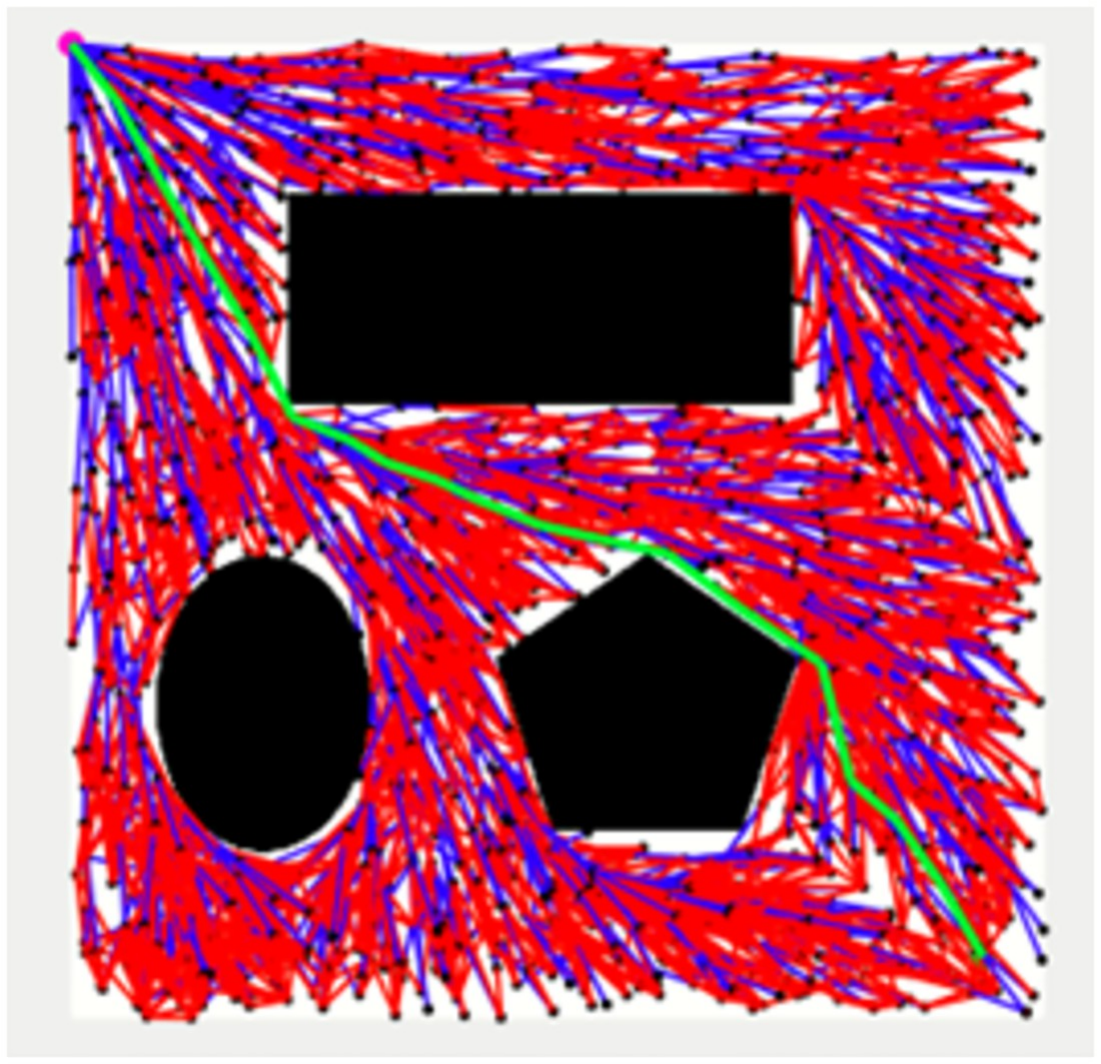
The result of RRT* for experiment 1.

**Fig 13:**
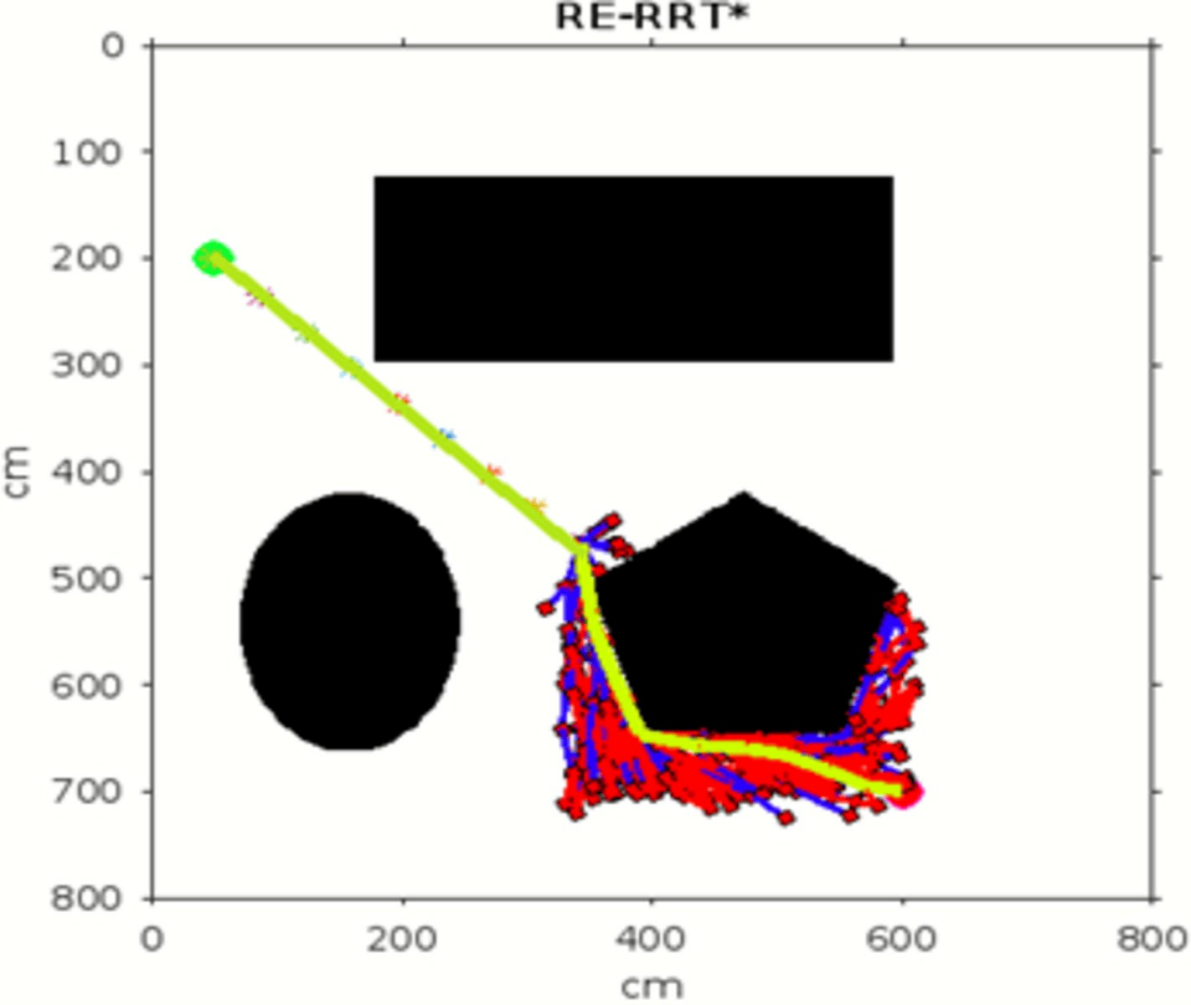
The result of RE-RRT* for experiment 1.

Figs 11–13 shows the schematic representation of simulated environment 1 with obstacles of different shapes. Fig 11 shows the result of RRT, where the blue lines show the random sampling over the whole map. The green irregular line is the path tracked by the RRT algorithm, which is not smooth. Fig 12 shows the result of the RRT* algorithm, which is the improved version of the RRT algorithm. The red lines show the rewired sampling. It generates a smoother path than RRT, but it takes a lot of time to execute the result because the sampling space is over the whole map. Fig 13 shows the result of the suggested method, the RE-RRT* algorithm. This is the improved method of the RRT* algorithm. We can see clearly in this picture how this method limits the random sampling and improves the execution time by 80% by limiting the sampling space only around the obstacle. As our vehicle started moving straight from the initial position towards the final position, when it reached the obstacle area, the RRT* algorithm was used to track the best route to pass the obstacle, and after tracking the path, the vehicle moved in a straight line towards the goal position.

**Fig 14:**
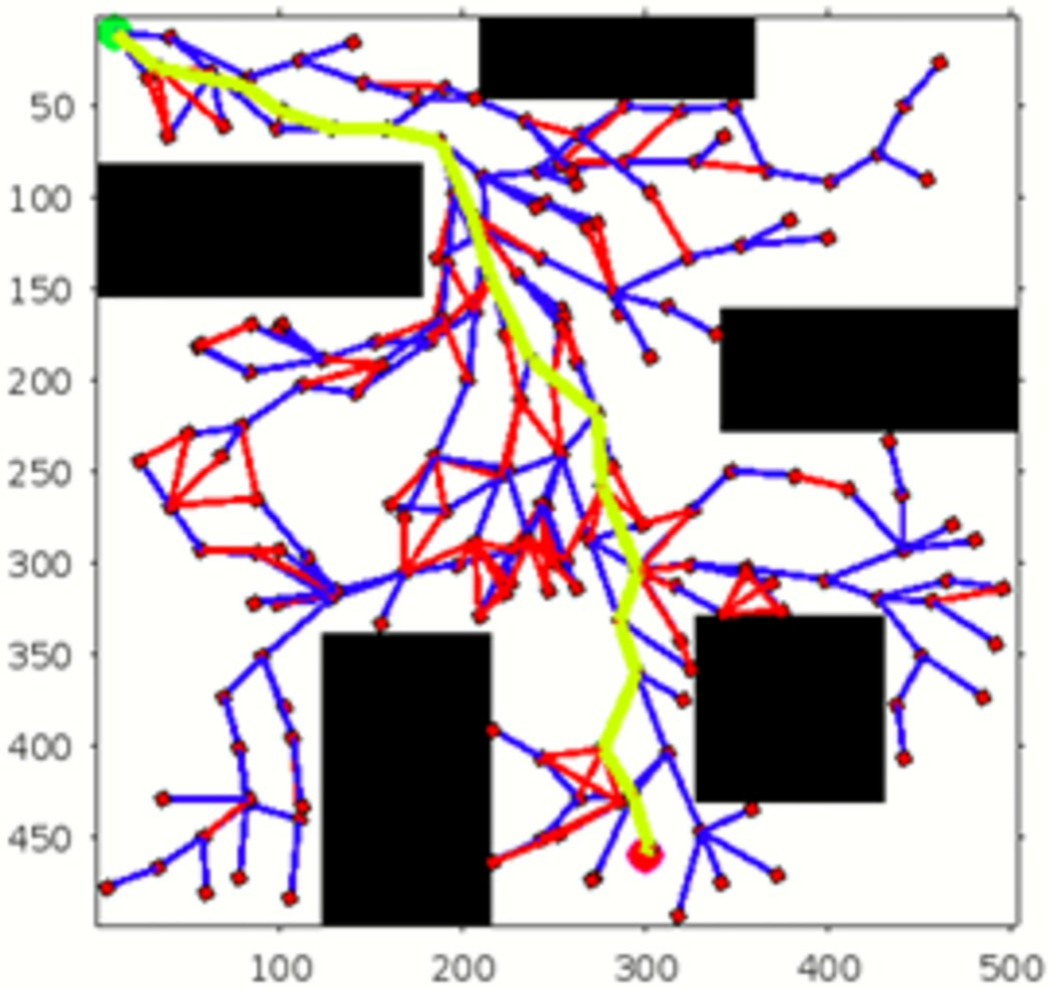
The result of RRT* for experiment 2.

**Fig 15:**
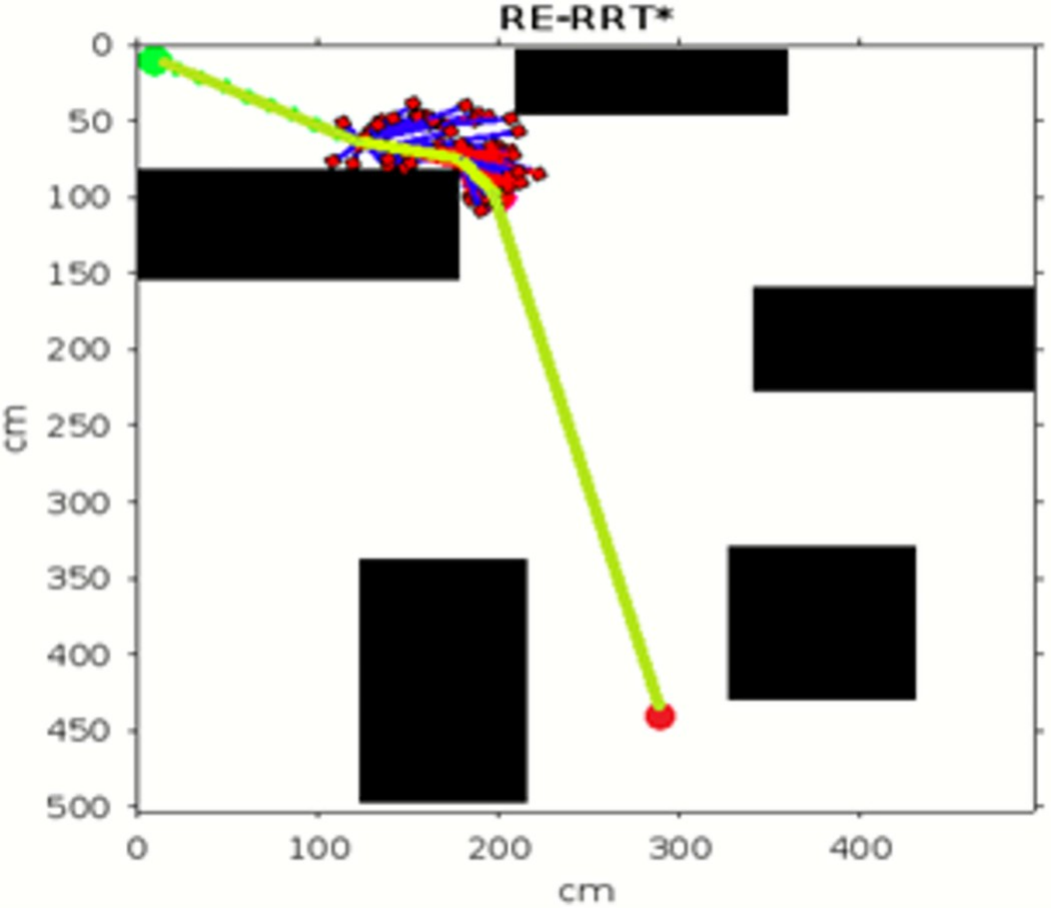
The result of RE-RRT* for experiment 2.

**Fig 16:**
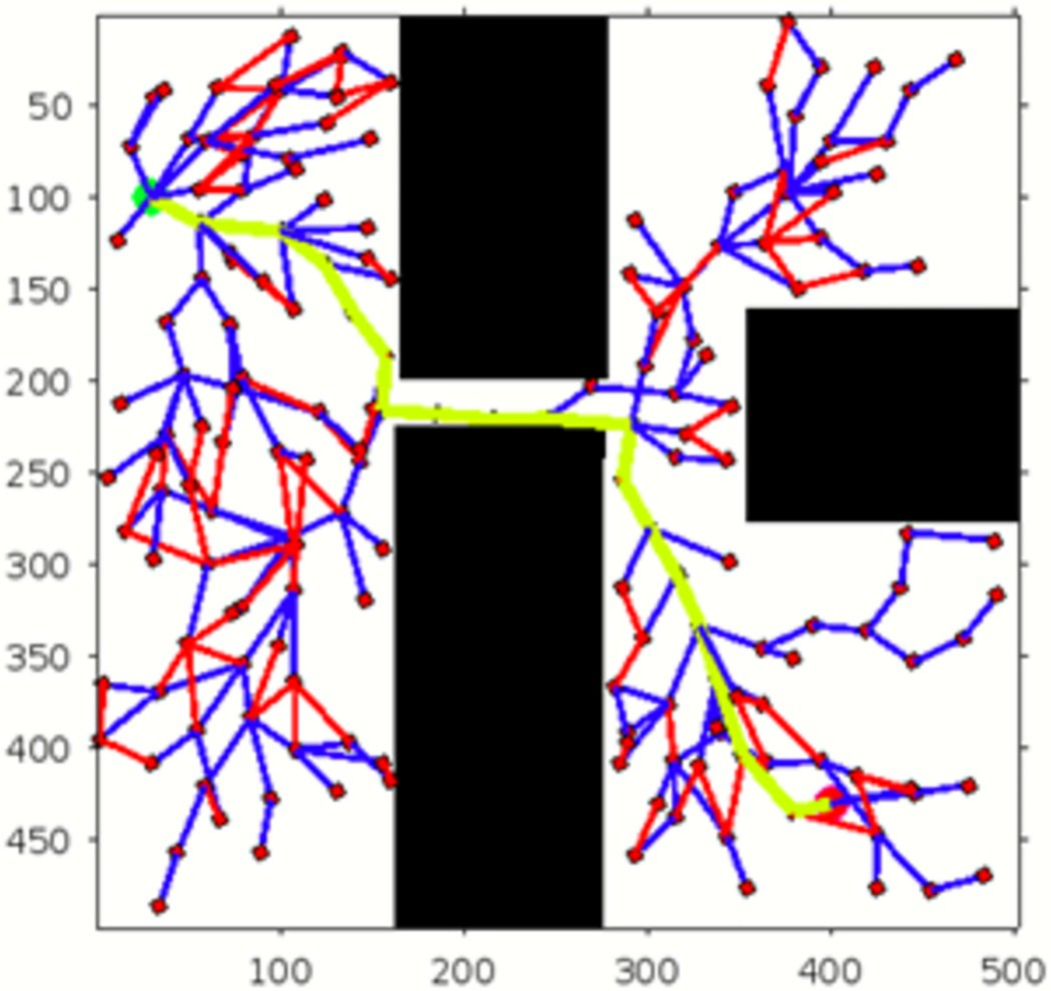
The 1st result of RRT* for experiment 3.

**Fig 17:**
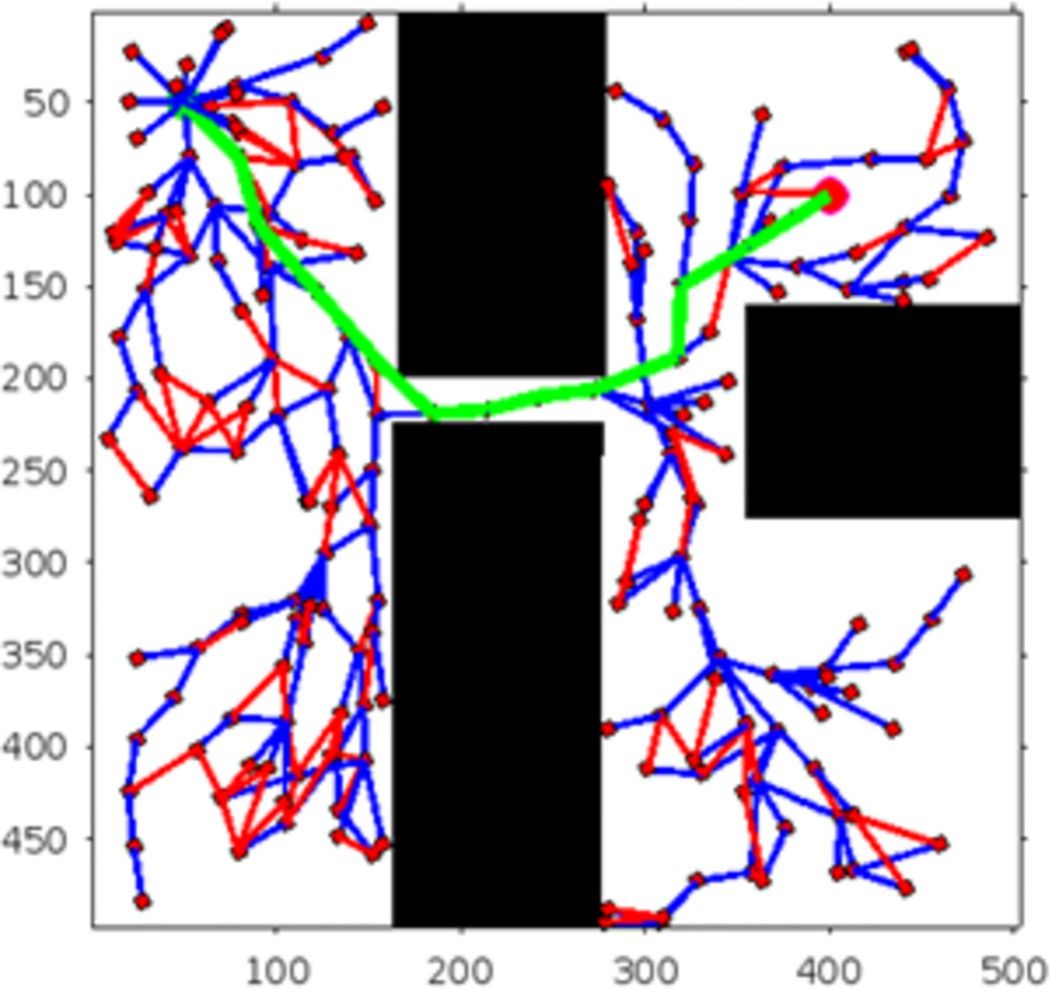
The 1st result of RE-RRT* for experiment 3.

**Fig 18:**
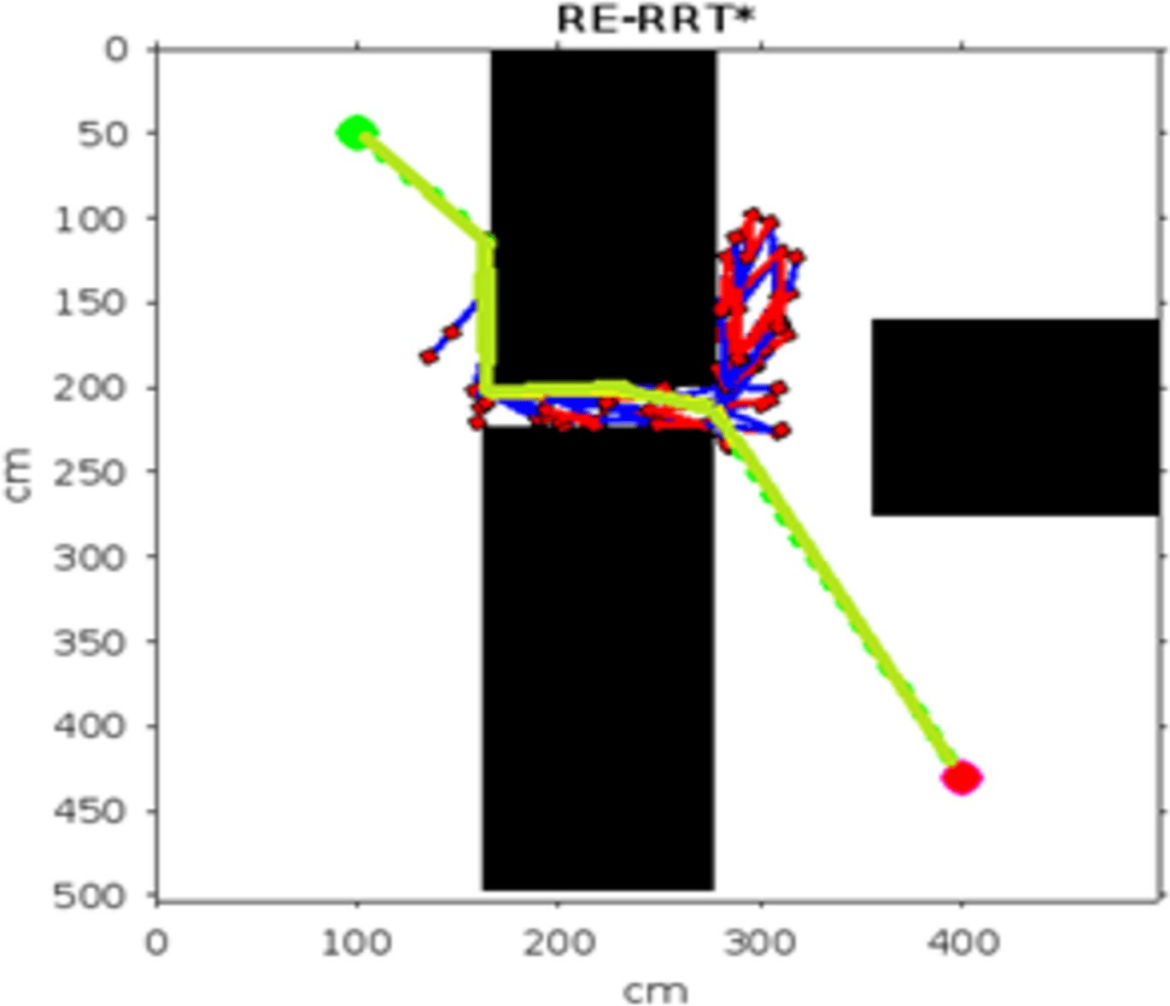
The 2nd result of RRT* for experiment 3.

**Fig 19:**
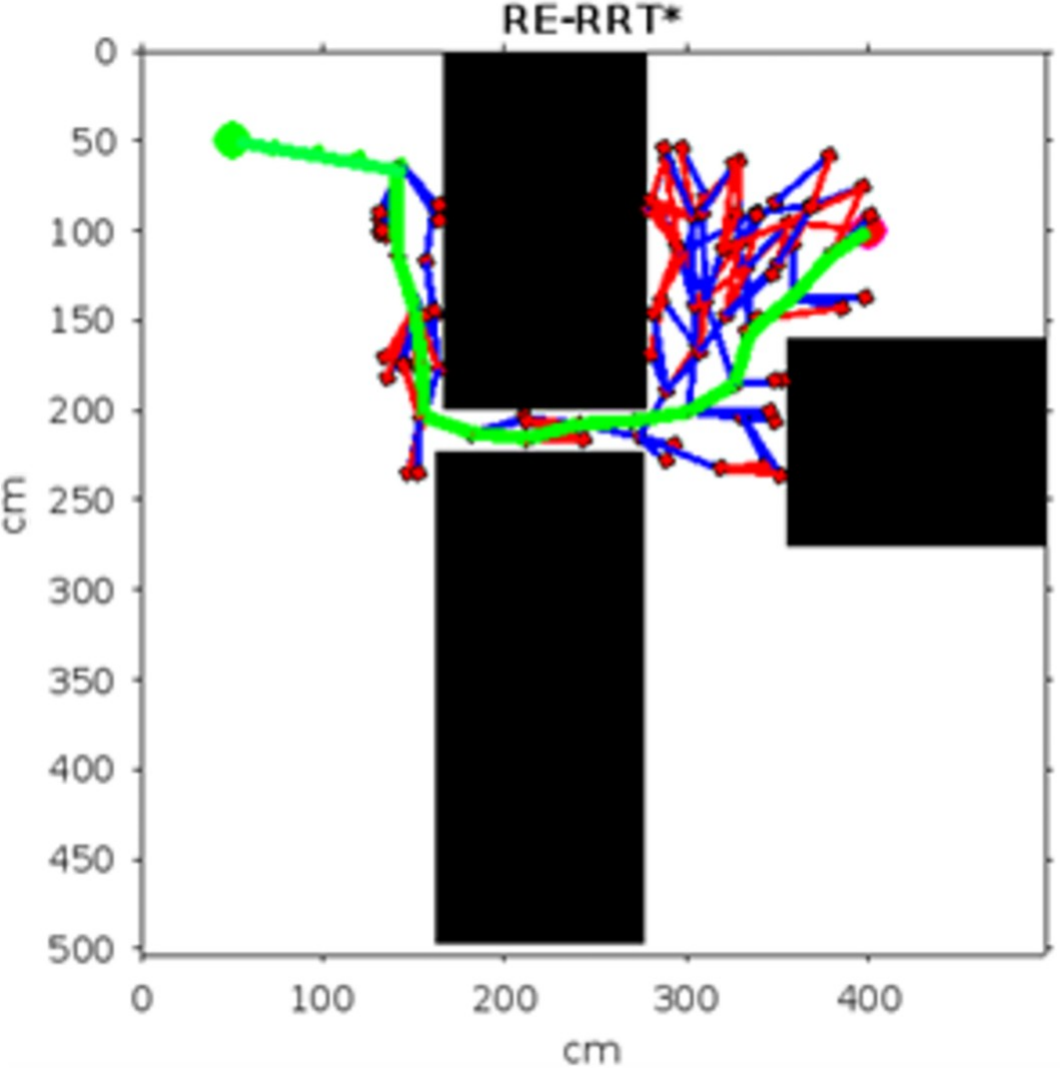
The 2nd result of RE-RRT* for experiment 3.

In the scenario of Environment 1 mentioned in Table 2, we can see that our method avoids a lot of unnecessary searches and speeds up the convergence rate. The basic RRT takes 26 seconds to converge to the goal position, while RRT* generates a smoother path than RRT but takes 3 to 4 minutes to rewire the node connections and yields a pruned output, which is not efficient. In contrast, our proposed RE-RRT* method takes only 11 seconds to converge to the goal position using the same parameters Fig 14 shows the schematic representation of RRT* for simulated environments 2 and Fig 15 shows the Re-RRT* for the same scenerio. Figs 16 and 17 shows simulation of environment 3 with a narrow path for RRt* and Figs 18 and 19 shows RE-RRT*. As we can see the difference between the results of RRT* and RE-RRT*. In RRT*, the nodes are generated randomly Over the whole map, it checks every node to see if it is optimal or not. So, this process makes the system slow and increases the convergence time to meet the desired goal. On the other hand, RE-RRT* minimizes the convergence rate even in a complex scenario to meet the desired goal by limiting random sampling only across the obstacles. Also, it reduces the number of turns through the pruning process. Tables 3–5 show the main evaluation indicators of RRT* and RE-RRT* path planning results for environments 2 and 3. In all cases, the RRT* generates pruned output in 1 to 2 minutes or 3 to 4 minutes, whereas the suggested RE-RRT* takes a maximum of 12 seconds and a minimum of 7 seconds to generate a smooth and pruned output and converge to the goal position.

**Table 2:** The main evaluation indicators of RRT, RRT* and RE-RRT* path planning results of environment 1.

Scenario	Total count/No of samplings	Distance (D)	Delete Index	Angle (*ϕ*)	Execution time (sec)
RRT	752	408	0	0.19	26
RRT*	738	256	270	-0.9	The first result in 22 sec pruned output takes 3-4min
RE-RRT*	282	241	66	-1.2	11

**Table 3:** The main evaluation indicators of RRT* and RE-RRT* path planning results of environment 2.

Scenario	Total count/No of samplings	Distance (D)	Delete Index	Angle (*ϕ*)	Execution time (sec)
RRT*	193	98	18	1.04	The first result in 22 sec pruned output takes 3-4min
RE-RRT*	76	54	3	0.6	11sec

**Table 4:** The main evaluation indicators of RRT* and RE-RRT* path planning results of environment 3 from Figs 16 and 18.

Scenario	Total count/No of samplings	Distance (D)	Delete Index	Angle (*ϕ*)	Execution time (sec)
RRT*	198	98	20	1.04	The first result in 22 sec pruned output takes 1-2min
RE-RRT*	80	61	6	0.6	11sec

**Table 5:** The main evaluation indicators of RRT* and RE-RRT* path planning results of environment 3 from Figs 17 and 19.

Scenario	Total count/No of samplings	Distance (D)	Delete Index	Angle (*ϕ*)	Execution time (sec)
RRT*	198	90	13	0.7	The first result in 14 sec pruned output takes 1 min
RE-RRT*	150	48	11	0.6	9sec

**Fig 20:**
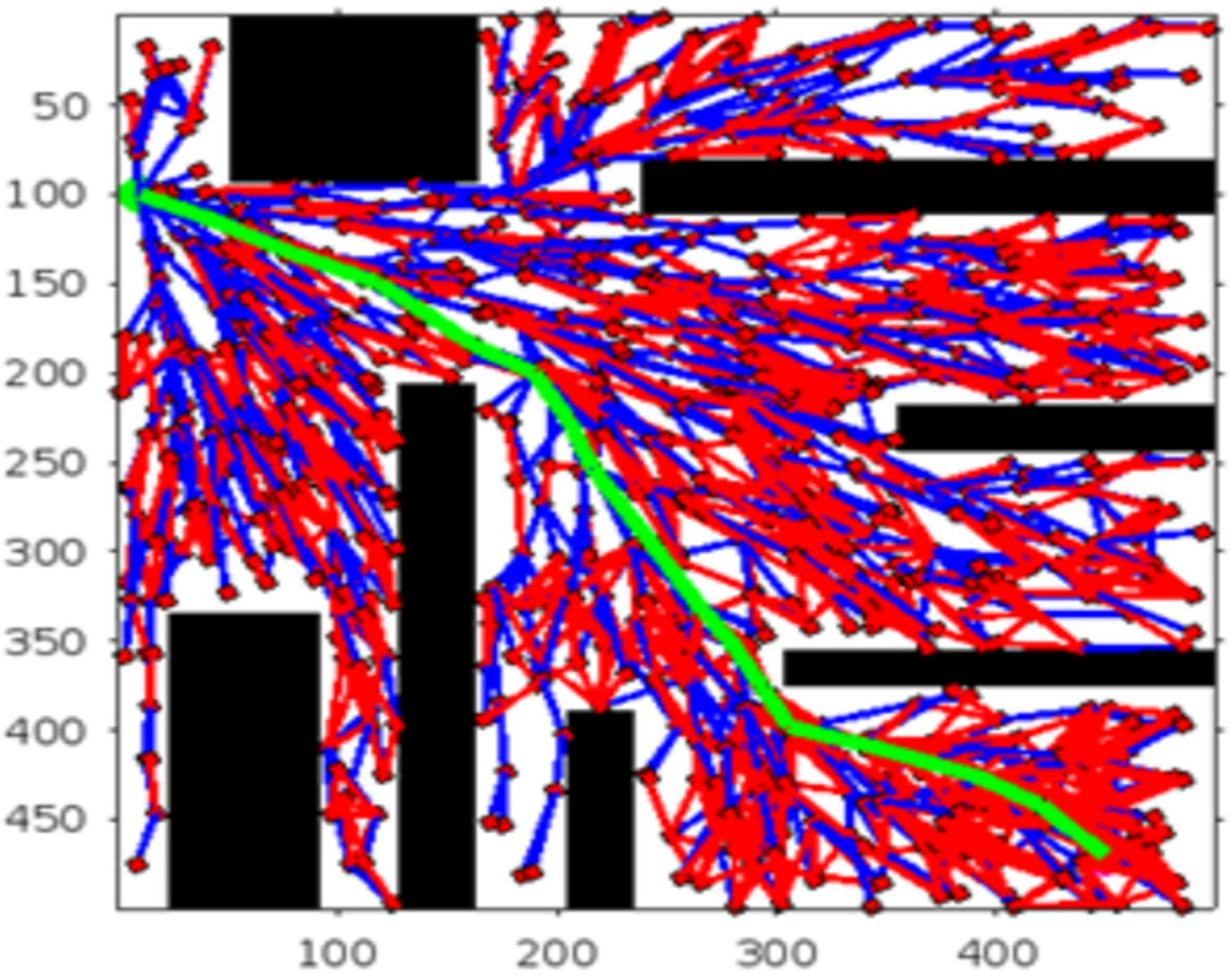
The result of RRT* for experiment 4.

**Fig 21:**
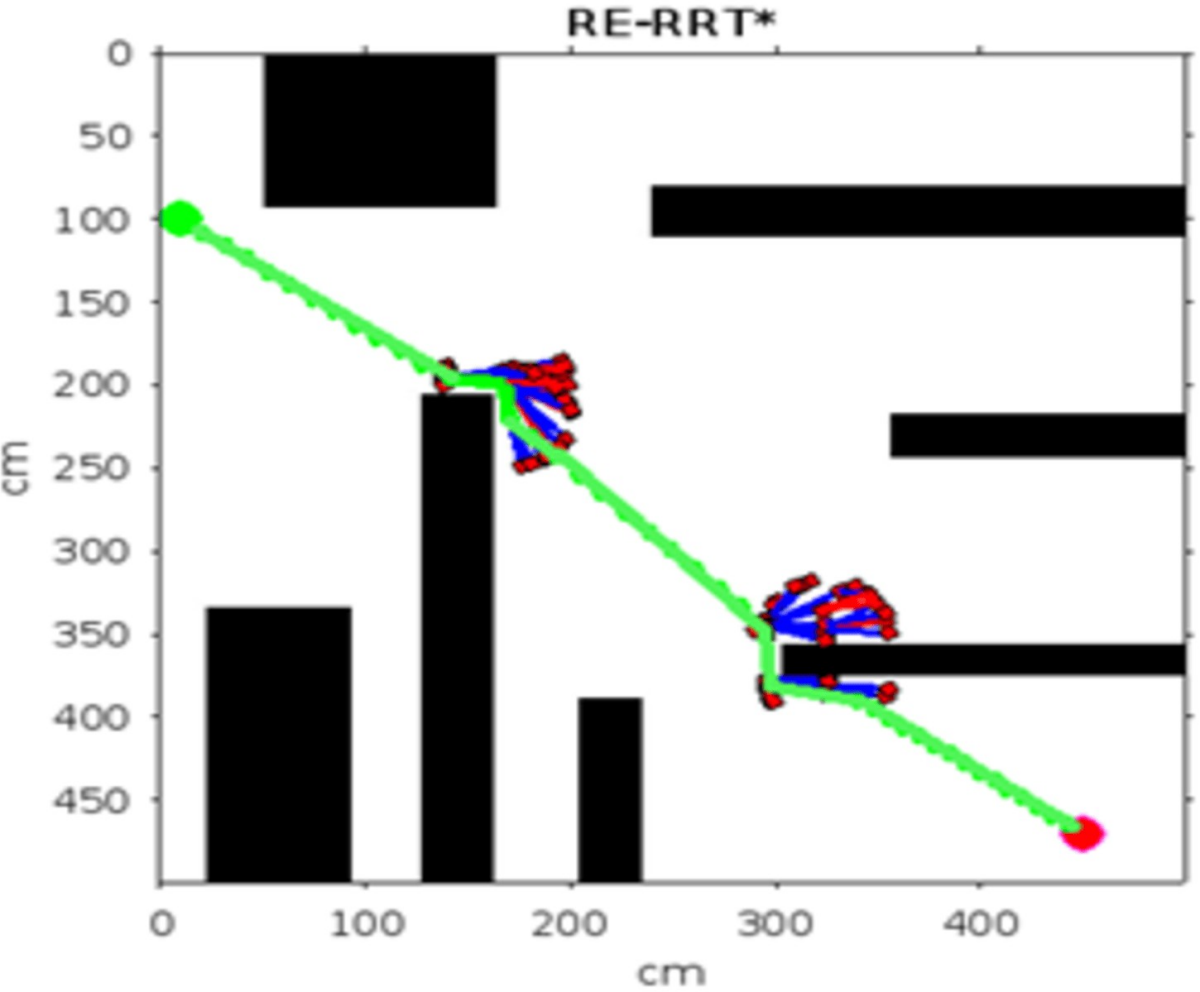
The result of RE-RRT* for experiment 4.

Figs 20 and 21. illustrates the schematic representation of simulated environment 4 with multiple obstacles on the way. Table 6 shows the comparison of the total count, distance, deleted index, theta (angle), and execution time between RRT* and RE-RRT*. RE-RRT* proves to be more efficient than the simple RRT* as it converges to the goal position in just 7 seconds.

**Table 6:** The main evaluation indicators of RRT* and RE-RRT* path planning results of environment 4.

Scenario	Total count/No of samplings	Distance (D)	Delete Index	Angle (*ϕ*)	Execution time (sec)
RRT*	427	98	281	1.6	The first result in 25 sec pruned output takes 3-4 min
RE-RRT*	75	45	12	0.9	7sec

Table 7 shows the comparison of our proposed method with existing algorithms. The RE-RRT* algorithm provides efficient path finding with low computational time (3.0 seconds) and a short path distance (250 meters), while maintaining a moderate cost. Neural RRT* assumes efficient path finding after training, achieving relatively lower computational time (2 seconds) and path distance (250 meters) with higher cost because the problem with Neural RRT* is that its dependent on training data. In complex environment, most of the time it is very difficult to train data for such scenarios. That’s why in comparison with our proposed algorithm, cost is lower and feasible for complex environment than Neural RRT*. On the other hand, RRT-Connect is slower, with higher computational time (4.0 seconds) and path distance (320 meters). Batch Informed Trees outperform RRT-Connect by offering faster computational time (3.5 seconds) and better path distance (270 meters).

**Table 7:** Comparison of RE-RRT* with existing algorithms.

Algorithm	Computational Time/(Sec)	Path Distance (m)	Convergence Rate (iterations)	Cost
RE-RRT*	3.0	250	50	Lower then the previous algorithm
Neural-RRT* [34]	2.0	250	60	Dependent on Data, In complex environment most of the time difficult to train data for such scenerios
RRT Connect [32]	4.0	320	1000	Higher due to full space exploration
Batch Informed Tree [35]	3.5	270	100	Lower due to batch processing
RRT* [28]	26	312	1000	Higher due to random exploration

## 5. Conclusion

To increase the speed and stability of determining the best path, we proposed the RE-RRT* algorithm, a novel RRT-based path planning technique. As a result, enhancements were made to random sampling, which is limited, which helps to avoid searching over the entire space. To increase the effectiveness of path planning even further, we developed the collision detection algorithm. The suggested RE-RRT* method has significant speed and stability improvements over the RRT and RRT* algorithms. For instance, as compared to the RRT method, the RE-RRT* algorithm dramatically reduced the average search time and its variance to locate a real path. At the same time, RE-RRT* is much faster than RRT* in terms of searching for a path that is close to optimal. As a result, our RE-RRT* method shows excellent promise in real-world motion planning applications. The simulation results in MATLAB showed that the algorithm has a fast convergence speed. Future efforts will concentrate on accelerating planning and adding more testing scenarios, such as testing with different types of robots or integration with sensor data for real-time adjustments.
